# PReS-FINAL-2281: The incidence of antiphospholipid antibodies in children with juvenile onset lupus erythematosus treated in Department of Pediatric Rheumatology, Institute of Rheumatology, Warsaw in the years 2002-2012

**DOI:** 10.1186/1546-0096-11-S2-P271

**Published:** 2013-12-05

**Authors:** A Gazda, B Kołodziejczyk, M Wierzbowska, P Gietka, I Szczygielska, L Rutkowska-Sak, J Ząbek, B Wojciechowska, A Kraska

**Affiliations:** 1 Department of Pediatric Rheumatology, Institute of Rheumatology, Warsaw, Poland; 2 Microbiology and Serology Department, Institute of Rheumatology, Warsaw, Poland; 3 Laboratory Diagnostic Department, Institute of Rheumatology, Warsaw, Poland; 4 Department of Ischaemic Disease, Institute of Cardiology, Warsaw, Poland

## Introduction

There were 54 patients with the juvenile onset of systemic lupus erythematosus included in the retrospective analysis, who were treated in the Institute of Rheumatology in Warsaw in the years 2002-2012. The average age of the onset of the illness in this group of patients was 12 years, the average time of observation was 3 years and 7 months.

## Objectives

In 20 patients (37%) high level of IgG anticardiolipin antibodies was discovered. High level of anticardiolipin antybodies IgM was discovered in 6 patients (11.1%). Lupus anticoagulant was determinated in 51 cases, in 17 was positive (31%). 8 patients had the tests for anti beta2GPI carried out and in 4 cases it was positive. In the group of 54 patients 22 (41%) presented prolonged activated partial thromboplastin time (aPTT). Thrombosis and thus antiphospholipid syndrome was confirmed only in 1 case, it was thrombosis of popliteal vein.

In 8 children the presence of two different antiphospholipid antibodies and prolonged aPTT were found with at least two hospitalizations. These children didn't have the symptoms of thrombosis during the time of observation (average 3.5 years) - Immunological APS.

In the last 10 years in our department were hospitalized also 2 children with antiphospholipid syndrome, they were diagnosed with Lupus-like disease. In the first case there was thrombosis of common femoral vein and deep femoral vein, the additional risk factor could be Legionella pneumophila pneumonia. The other child developed thrombosis of common iliac and external iliac vein, additionaly deficiency of XII factor was discovered. Among our Immunological APS patients there is also the girl with Libmann-Sachs endocarditis in the course of SLE, with aPTT, presence of lupus anticoagulant and anticardiolipin antibodies.

## Methods

Anticardiolipin antibodies were determinated by ELISA method, for isotype IgG positive was more then 4 SD (value 0.109), for isotype IgM more then 4 SD was 0.156. Lupus anticoagulant was determined due to recommendation of International Society of Thrombosis and Haemostasis. Anti beta 2 GPI antibodies were determined by standardised ELISA method.

## Results

There were two groups in which the clinical symptoms and laboratory investigations were compared: the first group consisted of the patients (24 people) who never had antiphospholipid antybodies (aPL ab), in the second group there were 20 patients who presented presence of aPL ab, some of them had also prolonged aPTT. The incidence of laboratory markers of antiphospholipid syndrome correlated with significantly higher ESR (33 versus 19), higher level of antinuclear antibodies (1769 versus 550), but the average values SLEDAI index were comparable (10.4 versus 9.24). Among other symptoms, the thrombocytopenia, proteinuria and vasculitis were significantly more often in the group with laboratory markers of antiphosphoslipid syndrome.

## Conclusion

In spite of the high incidence of immunological markers of antiphosphoslipid syndrome thrombosis and thus antiphospholipid syndrome is rare. The pathogenetic role of antiphospholipid antibodies need more investigations.

## Disclosure of interest

None declared.

